# Synapse Clusters Are Preferentially Formed by Synapses with Large Recycling Pool Sizes

**DOI:** 10.1371/journal.pone.0013514

**Published:** 2010-10-20

**Authors:** Oliver Welzel, Carsten H. Tischbirek, Jasmin Jung, Eva M. Kohler, Alexei Svetlitchny, Andreas W. Henkel, Johannes Kornhuber, Teja W. Groemer

**Affiliations:** Department of Psychiatry and Psychotherapy, University of Erlangen-Nuremberg, Erlangen, Germany; University of Nebraska Medical Center, United States of America

## Abstract

Synapses are distributed heterogeneously in neural networks. The relationship between the spatial arrangement of synapses and an individual synapse's structural and functional features remains to be elucidated. Here, we examined the influence of the number of adjacent synapses on individual synaptic recycling pool sizes. When measuring the discharge of the styryl dye FM1–43 from electrically stimulated synapses in rat hippocampal tissue cultures, a strong positive correlation between the number of neighbouring synapses and recycling vesicle pool sizes was observed. Accordingly, vesicle-rich synapses were found to preferentially reside next to neighbours with large recycling pool sizes. Although these synapses with large recycling pool sizes were rare, they were densely arranged and thus exhibited a high amount of release per volume. To consolidate these findings, functional terminals were marked by live-cell antibody staining with anti-synaptotagmin-1-cypHer or overexpression of synaptopHluorin. Analysis of synapse distributions in these systems confirmed the results obtained with FM 1–43. Our findings support the idea that clustering of synapses with large recycling pool sizes is a distinct developmental feature of newly formed neural networks and may contribute to functional plasticity.

## Introduction

Current research on synaptic transmission seeks to understand the complex regulation of individual synapses within neural networks. Especially the modification of synaptic connections as a crucial element of structural plasticity and long-term memory has been in the focus of intense research [1]. A multitude of influences is known to lead to the rewiring of neural circuits via synapse formation and elimination. For instance, decreased neuronal activity results in the establishment of new, less clustered synaptic connections [2], [3], [4], [5] and strengthening of inactive synapses [6].

Besides these structural considerations, functional parameters of synapse populations such as spontaneous activity [7], time-course of endocytosis [8], release probability [9], [10], analysis of different synaptic vesicle pools [11], [12], [13] and single synaptic vesicle exocytosis [14], [15], [16], [17] have been examined. In these studies, the distribution of features such as vesicle pool sizes or release probabilities was typically skewed positively with a predominance of lower values [6], [9], [10], [18], [19] and in some cases approximated by a Γ density function [9], [10].

Interestingly, morphological features have been shown to be relevant for the modification of individual synaptic connections. For example, the release probability between neighbouring synapses is highly correlated [9], [10], and also decreases with the number of synapses formed by an axon [10]. A study in L2/3 cortical cells has suggested that synapses onto the same postsynaptic target adopt the same efficacy of neurotransmitter release regardless of their position in the dendritic tree [20]. In addition, it was shown that nearby synapses are functionally connected by sharing of recycling synaptic vesicles between presynaptic boutons [21]. Recently, experimental evidence in acute hippocampal slices also showed that vesicles trafficked across multiple terminals were readily available to all neighbouring synapses [22].

To extend the link between morphological observations and synaptic function, in this study the release characteristics of individual synapses were investigated with regard to their spatial arrangement. This was made possible by the fact that with fluorescence microscopy other than with electron microscopy the number of vesicles in a terminal can be assessed in a highly parallelized approach ([9]. The environments of a high number of synapses (more than 30000 in our study) were explored in the well investigated rat hippocampal tissue culture system [6], [9], [15], [18], [23], [24], [25] with synapse parameters comparable to histological preparations [26], acute slices [22], [27] and *in vivo* measurements [2], and the vesicle recycling of individual boutons was analyzed with the established styryl FM dyes [28].

Within the heterogeneous spatial distribution of synapses, we identified areas with particularly high numbers of boutons as synapse clusters, which were preferentially composed of synapses with large recycling pool sizes. These results were confirmed with two independent functional synapse labelling systems, i.e. the uptake of the CypHer 5 labelled antibody against synaptotagmin1 (αSyt1-cypHer) [29], [30] and synaptobrevin-2-pHluorin (spH) [31]. Taken together, we describe a novel connection between the release characteristics of individual synapses and their number of synaptic neighbours in a defined environment.

## Materials and Methods

### Ethics statement

All animals were handled in strict accordance with good animal practice as defined by the guidelines of the State of Bavaria, and all animal work was approved by the Kollegiales Leitungsgremium of the Franz-Penzoldt Zentrum, Erlangen (reference number TS-1/10).

### Cell culture and transfection

Hippocampal neuronal cultures were prepared from one to four days old Wistar rats (Charles River, USA). Briefly, newborn rats were sacrificed by decapitation in accordance with the guidelines of the State of Bavaria. Hippocampi were removed from the brain and transferred into ice cold Hank's salt solution, and the dentate gyrus was cut away. After digestion with trypsin (5 mg ml^−1^) cells were triturated mechanically and plated in MEM medium, supplemented with 10% fetal calf serum and 2% B27 Supplement (all from Invitrogen, Taufkirchen). If required, neurons were transfected with synaptopHluorin under control of a synapsin promoter [32] on DIV3 with a modified calcium phosphate method as described [33]. In brief, the culture medium was removed and replaced with Neurobasal A (Invitrogen, Taufkirchen). The calcium phosphate/DNA precipitate was allowed to form in BBS buffer (pH 7.05) for 30 min, then cells were transfected dropwise with the precipitate in Neurobasal A and incubated for 30 min prior to washing with HBSS (Invitrogen, Taufkirchen). There was no apparent long-term toxicity to the cells, as assessed by lactate dehydrogenase assays (CytoTox96® Non-Radioactive Cytotoxicity Assay, Promega, Mannheim), data not shown. Experiments were performed between 18 and 21 days *in vitro*.

### Imaging

Experiments were conducted at room temperature on a Nikon TI-Eclipse inverted microscope equipped with a 60×, 1.2 NA water immersion objective and Perfect Focus System™. Fluorescent dyes were excited by a Nikon Intensilight C-HGFI through excitation filters centred at 482 nm and 640 nm using dichroic longpass mirrors (cut-off wavelength 500 nm and 650 nm), respectively. The emitted light passed emission band-pass filters ranging from 500 nm–550 nm and 650 nm–700 nm, respectively (Semrock, Rochester) and was projected onto a cooled EM-CCD camera (iXon^EM^ DU-885, Andor).

Cover slips were placed into a perfusion chamber (volume = 500 µl) containing extracellular medium containing (in mM): 144 NaCl, 2.5 KCl, 2.5 CaCl_2_, 2.5 MgCl_2_, 10 Glucose, 10 Hepes and pH 7.4. Synaptic boutons were stimulated by electric field stimulation (platinum electrodes, 10 mm spacing, 1 ms pulses of 50 mA and alternating polarity); 10 µM 6-cyano-7-nitroquinoxaline-2,3-dione (CNQX, Tocris Bioscience) and 50 µM D-amino-5-phosphonovaleric acid (D,L-AP5, Tocris Bioscience) were added to prevent recurrent activity.

Recycled synaptic vesicles were labelled with FM 1–43 (Invitrogen, Karlsruhe). To stain the total recycling pool nerve terminals were loaded with 1200 action potentials (APs) at 40 Hz [19], [34], [35] using 2.5 µM FM 1–43. The dye was allowed to remain on the cells for 60 s after cessation of the stimulus to permit complete compensatory endocytosis, and was subsequently removed during a 7 minute period with 8 complete exchanges of the solution. The loaded boutons were then stimulated with 600 APs at 30 Hz to evoke exocytosis. To obtain a measure for the total amount of loaded vesicles, we completely destained boutons using a twofold stimulation with 900 APs at 30 Hz [12]. Images were recorded with 200 ms exposure time at 0.5 Hz frame rate and 500 ms exposure time at 2 Hz frame rate for the stimulation with 600 APs, respectively. The frame rate was 0.25 Hz for the double total destain stimulation procedure with 900 APs. For the measurement of quantum intensities, we performed subtotal staining experiments (20 APs at 0.5 Hz) as described [9]. Loaded boutons were completely destained with two consecutive stimulations of 300 APs and 600 APs at 5 Hz. Images were recorded with 500 ms exposure time at 0.05 Hz frame rate.

For the anti-synaptotagmin-1-cypHer experiments cultured dispersed hippocampal neurons were incubated for 0.5 h to 1 h with 0.6 µg of CypHer™5E labelled anti-synaptotagmin1 antibody (αSyt1-cypHer) (Synaptic Systems, Goettingen) in extracellular medium to stain synaptic boutons. To identify active synapses, cells were electrically stimulated with 1200 AP at 40 Hz. Images were recorded with 2000 ms exposure time at a framerate of 0.33 Hz.

In the synaptopHluorin experiments boutons were stimulated with 600 APs at 30 Hz. Images were recorded with 100 ms exposure time at a framerate of 0.5 Hz. For all experiments camera binning was 2×2 and resulting image stacks were converted into tagged image file format (TIFF).

### Image analysis

**Figure 1:**
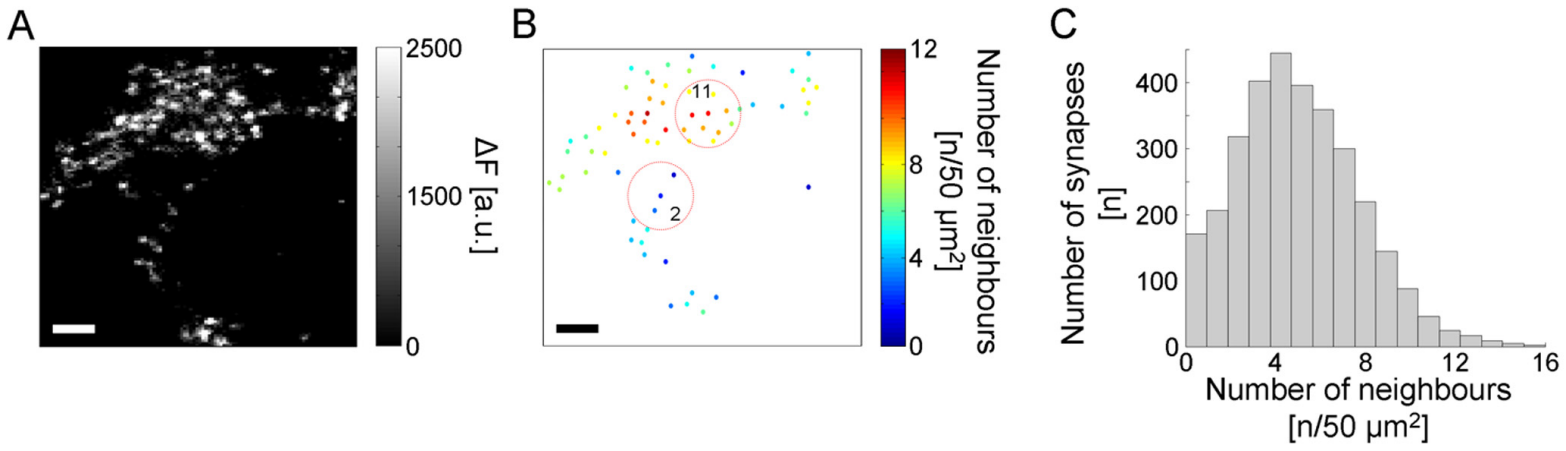
Distribution of the number of neighbouring synapses in cultured hippocampal neurons. **A**, Difference-image of images before and after electrical stimulation of FM 1–43 labelled neurons in a representative experiment. **B**, Analytical image calculated from **A**. Positions of automatically detected synapses are represented by dots and colour-coded according to their number of neighbouring synapses in a 50 µm^2^ environment. Environments of two representative synapses with two and eleven neighbours are highlighted, respectively. **C**, Distribution of the number of synapse neighbours (N = 5, n = 3149). (Scale bars 4 µm).

All image and data analysis was performed using custom-written routines in MATLAB (The MathWorks, Inc., Natick). The mean background determined from the intensity histogram of recorded image stacks was subtracted [36] and the resulting image stacks were used to automatically define peak regions of interest of synaptic bouton size [37], where AP-evoked fluorescence decrease (FM 1–43 and anti-synaptotagmin-1-cypHer) or increase (synaptopHluorin) occurred in difference images. The average for all regions of interest was calculated for each image to obtain fluorescence intensity profiles. For the determination of the total pool size ΔF_tot_ the mean of five values before the onset of the stimulus was subtracted from the mean of five values after the total destain period. The absolute fluorescence decrease ΔF (FM 1–43 and anti-synaptotagmin-1-cypHer) or increase (synaptopHluorin) was calculated as the difference of the mean of three (anti-synaptotagmin-1-cypHer) or five (FM 1–43 and synaptopHluorin) values before the first stimulation and the mean of three or five values after the end of the first stimulus with 600 APs or 1200 APs, respectively. The number of neighbours was determined by counting the detected spots in a circular region with a diameter of 30 pixels (pixel size *p_s_* = 267 nm at binning 2×2), which correspond to a diameter of 8 µm, around each spot (see Figure 1B) or approximately 50 µm^2^. For the determination of the synapse diameter each spot was fitted by a rotation symmetric gaussian profile: 
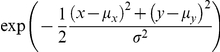
, where x and y are the image coordinates, μ_x_ and μ_y_ are the center coordinates of each spot and σ is the standard deviation. The area around each spot chosen for the fit was 10×10 pixels. The tolerance for the used unconstrained nonlinear optimization was 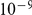 and results of the fit with a coefficient of determination below 0.8 were discarded. The diameter of each synapse was calculated as the full width at half maximum, using the following equation: 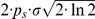, with p_s_ as the pixel size obtained from physical camera pixel size and magnification of the used objective.

### Definitions of classes of recycling pool sizes

When a continuous distribution is described, it might be useful to introduce the terms “high values”, “low values” and “average values” in order to emphasize differences in their individual properties. Accordingly, we discriminated between “synapses with large recycling pool sizes” and “synapses with small recycling pool sizes” that we defined as synapses with the highest or lowest 10% of recycling vesicle numbers as well as “synapses with average recycling pool sizes” (median +/− 5%) respectively.

### Statistical analysis

Statistical analysis was performed by MATLAB (The MathWorks, Inc., Natick). Differences in the mean or median of data points (e.g. see Figure S1D) were tested using a Wilcoxon rank sum test or two-sample t-test, respectively. If required, the first bin was compared with all other bins. Levels of significance p (p-values) are indicated as follows: * p<0.05, ** p<0.01 and *** p<0.001. Furthermore Spearman's rank correlation coefficient *ρ* between two parameters was calculated. To clarify the statistical results obtained from Wilcoxon rank sum test and Spearman's rank correlation coefficient *ρ*, we used the effect size *d* [38] as a sample size independent measure, which specifies the quantity of an effect. We calculated Cohens *d* between the first and the last bin by: 
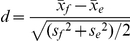
, where 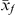 and 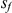 are the mean and the standard deviation of the first bin and 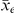 and 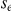 are the estimated mean and standard deviation of the last bin, respectively.

## Results

### The number of synaptic neighbours is distributed heterogeneously in cultured hippocampal neurons

Complete staining of the recycling vesicle pool using styryl dye FM 1–43 [19], [34], [35] resulted in punctuated distribution of fluorescence [8], [39]. Subtraction of fluorescence levels after total destaining (see Materials and Methods) resulted in a difference-image, in which the intensity of spots corresponded to the stimulation-dependent loss of fluorescence and thereby marked active synaptic terminals (see Figure 1A). The variation of synapse density was analysed by automatically detecting the synapses and their number of neighbours in an arbitrarily defined environment that we chose to be a circular region (50µm^2^) around each detected synapse (see Figure 1B). The results were conserved when testing different environments between 25 µm^2^ and 100 µm^2^ (data not shown). In analogy with the positively skewed distribution of the recycling pool size and release probabilities of synapses [9] the number of neighbours was heterogeneously distributed (see Figure 1C). We therefore observed regions with a high number of neighbours, which are commonly termed synapse clusters [2], [5]. In accordance with previous studies [2], [5], we refrained from any threshold definition of synapse clusters. Thus when used below, the term “synapse clusters” describes environments in which the density of synapses is relatively high when compared to other environments within the same experiment without relying on a threshold.

### Synapse clusters are formed by synapses with large recycling pool sizes

Next we wanted to clarify if synapses localized in clusters exhibited common or different synaptic features compared to boutons surrounded by low numbers of synaptic neighbours. For better inter-experimental comparison we replaced arbitrary fluorescent units with the number of synaptic vesicles in the recycling pool. This was achieved by determining the fluorescence of a single vesicle by performing subtotal staining experiments with 20 AP at 10 Hz (n = 613; N = 3) as described [9]. To analyze the quantisation, we applied multi-Gaussian fits to the intensity histogram of each experiment with the prerequisite of constant half-widths for all Gaussians allowing for varying amplitudes and means. We found a clear quantisation with almost constant centre-to-centre differences of 20.02±1.91 arbitrary units (Figure S1A).

**Figure 2:**
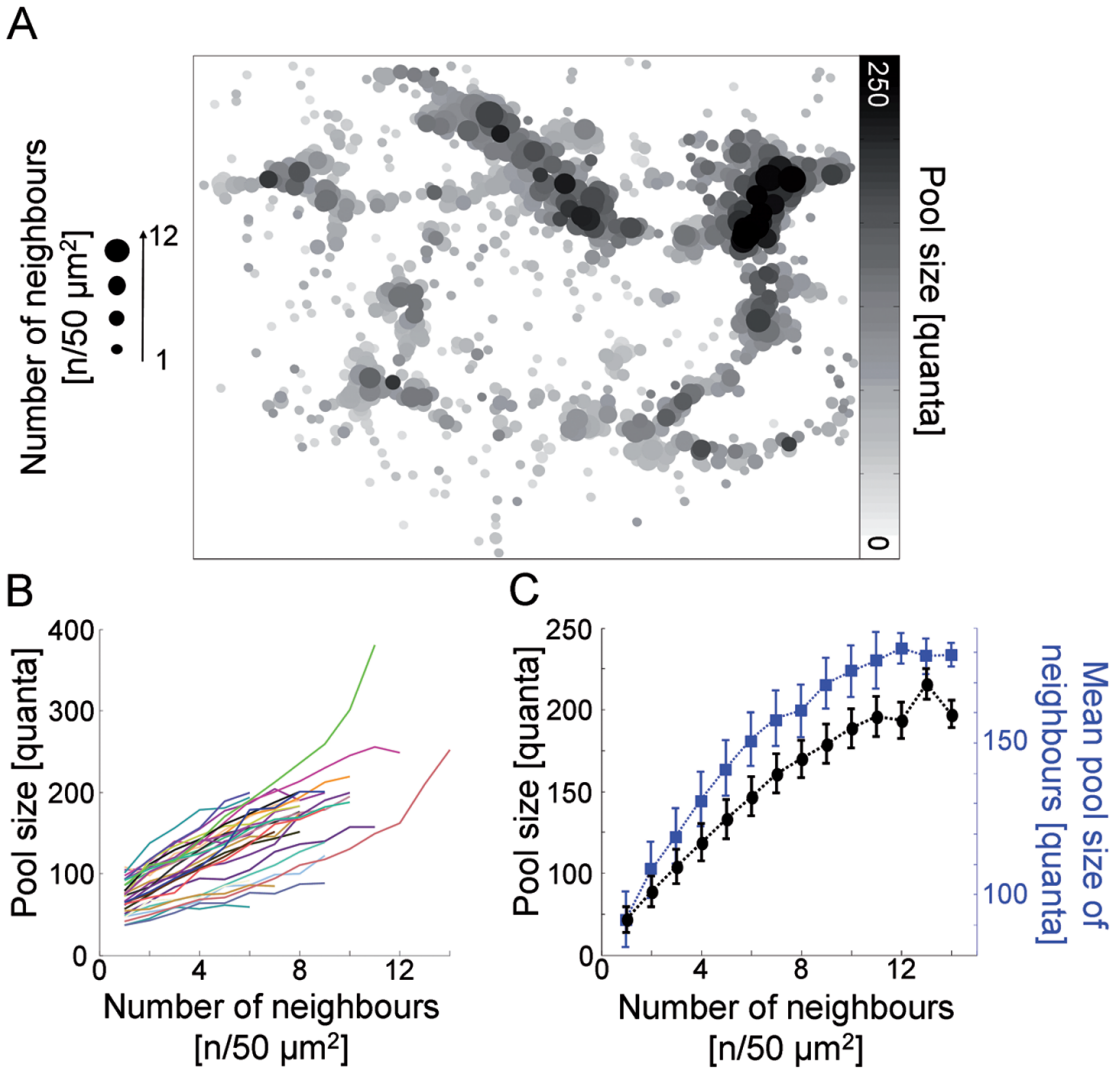
Relation of recycling pool size and number of neighbours. **A**, Analytical image calculated from a representative FM 1–43 turnover experiment. Each detected synapse is marked by a filled circle, which is gray-shaded according to its recycling pool size and size-coded for its number of neighbours in a 50 µm^2^ environment. (For the original difference image of images before and after electrical stimulation see Figure S2). **B**, Relation of recycling pool sizes and neighbour numbers in 30 independent FM 1–43 turnover experiments. Each experiment is represented by an individually coloured line. There was a strong correlation between pool size and number of neighbours that was conserved in all individual experiments (N = 30; mean Spearman's *ρ* = 0.97±0.03, p<0.01). **C**, Summary graph of **B**. Black circles: Relation between number of neighbours and vesicle pool sizes (Cohens *d* = 4.76; Spearman's *ρ* = 0.97, p<0.001). Blue squares: Relation between the number of neighbours and the mean pool size of their neighbouring synapses (Cohens *d* = 2.30; Spearman's *ρ* = 0.95, p<0.001). Error bars indicate standard error of the mean (N = 30, n = 22528).

When analyzing the recycling pool size and the number of neighbours of each synapse, we observed that synapses located within synapse clusters contained significantly more recycling vesicles (Figure 2A,C; Wilcoxon rank sum test: p<0.001). Thus, when correlating number of neighbours and recycling pool size, a strong correlation between these parameters was observed in 30 independent experiments (Figure 2B). In principle, this correlation could arise from appraisal artefacts as synapses located in close proximity to each other are more likely to overlap. However, the correlation was still observed for synapses with low number of neighbours. The difference of recycling pool sizes between synapses with, for example, a single neighbour and those with three neighbours is already highly significant (Wilcoxon rank sum test: p<0.001). These synapses reside in sparsely populated areas of the culture and their overlap on the acquired image is unlikely.

A considerable variance in the maximal number of neighbours as well as the recycling pool size was found in each experiment, which is likely due to the individual outgrowth of neurons on each coverslip. The relation between recycling pool size and number of neighbours, however, was conserved in all individual experiments (Spearman's *ρ* = 0.97±0.03, p<0.01) and was limited by the maximal recycling pool size, and thus saturation was observed with an increasing number of neighbours (see Figure 2C). Furthermore, in the summary of all experiments the correlation between recycling pool size and number of neighbours had a high effect size (Cohens *d* of 4.76) [38]. This, however, did not prove that synapses with many neighbours are located next to synapses with large vesicle pools. We thus measured the mean pool size of neighbours around the individual synapses and found that an increasing number of neighbours correlated with a larger mean vesicle pool sizes of the surrounding synapses (Cohens *d* = 2.30; Spearman's *ρ* = 0.95, p<0.001). We found that synapse clusters consisted of synapses with large recycling pools, which is not necessarily identical to the finding that synapses with large recycling pools are surrounded by a high number of neighbours.

### Comparison of synaptic features in the investigated system to findings of previous studies

In order to validate our study we wanted to know if our culture system shared commonly described characteristics. We thus tested the parameters known to be heterogeneously distributed among individual synapses, namely recycling pool size, correlation of exocytosed quanta with recycling pool size, release probability and synapse diameter on their compatibility to previous reports.

We first tested if the positively skewed recycling pool size distribution [9] was preserved in our culture system. The histogram of recycling pool sizes showed a clear positive skew and thus relatively few synapses with large vesicle pools (Figure S1B). Additionally, in accordance with previous studies [9], [18], we found that the number of fused vesicles upon a stimulus of 600 APs at 30 Hz was linearly correlated to the pool size. (Figure S1B; Cohens *d* = 9.84; Spearman's *ρ* = 0.96, p<0.001). The mean value of the recycling pool size (137 vesicles) was also similar to previously published data (127 vesicles) in a typical hippocampal tissue culture [10], [18]. Synapse sizes (estimated from 2D Gaussian fits of the diameter of the total stained vesicle population of individual boutons) increased with the recycling pool size [40] (Cohens *d* = 2.49; Spearman's *ρ* = 0.51, p<0.001, Figure S1D). Mean and standard deviation of synapse diameters were 882.09 nm±249.92 nm and again in good agreement with previous ultrastructural studies [26], [41], [42]. Furthermore, we determined the release probability following stimulation with 20 APs. The distribution of release probability *p* is continuous and the histogram was skewed positively with a median of 0.19. A fit with Γ density function [9] yielded λ = 8.32 (Figure S1C) and was again in the range of previous works [9], [10]. Taken together, these results showed that the properties of the preparation used here were comparable to previous reports and validated that synapses with large recycling pool sizes with high release probabilities and diameters can preferentially be found in synapse clusters.

### Comparison of the spatial arrangement of synapses in three different functional labelling assays

In order to exclude that synapse clusters of vesicle rich synapses are a phenomenon of styryl-dye labelling we tested sets of experiments in which we had used an antibody based functional labelling system as well as overexpression experiments for their compatibility with the results from FM-dye labelling.

For live-cell antibody labelling of functional terminals we used a monoclonal antibody directed against the intra-vesicular domain of synaptotagmin-1. Synaptotagmin-1 becomes accessible on the cell surface after synaptic vesicle exocytosis and can be labelled with an antibody, which is then internalized when the vesicles are retrieved [43]. This monoclonal antibody was coupled to the pH-sensitive Cy-5 dye variant CypHer 5 (αSyt1-CypHer) [29], [30]. This approach selectively labels functional terminals as CypHer 5 fluoresces in the acidic milieu of synaptic vesicles (pH 5.5) but not when exposed to extracellular pH (pH 7.4). Besides the selective labelling of functional terminals by their antibody uptake it enables visualization of synaptic exo- and endocytosis cycles according to the stimulation dependent change in fluorescence. Thus this pH-dependent probe provides a good measure for the number of released vesicles which in turn highly correlates with the size of the recycling pool (Figure S1B).

**Figure 3:**
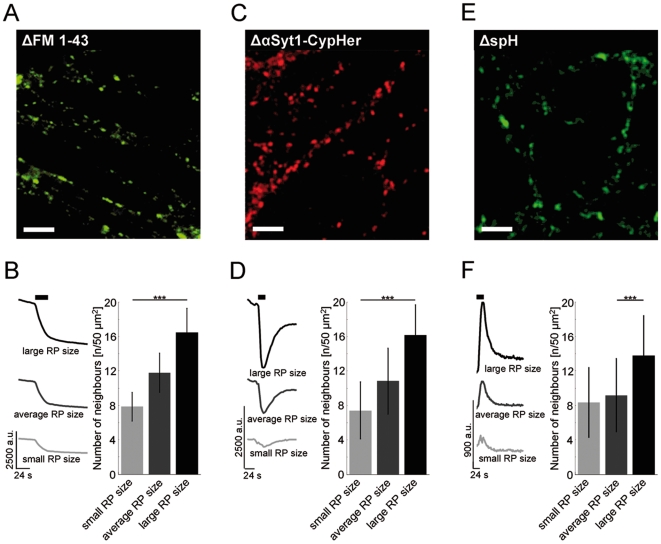
Neighbour-numbers of synapses of different pool sizes in three different functional fluorescence labelling approaches. **A**, **C**, **E**, Representative fluorescence difference images of (**A**) FM 1–43, (**C**) αSyt1-CypHer, and (**E**) synaptopHluorin before and after electrical stimulation. **B**, **D**, **F**, Synapses were grouped according to their recycling pool size (see Materials and Methods). Fluorescence time courses and neighbour-numbers of synapses with small, average and large recycling pool (RP) sizes in FM 1–43 (**B**) (N = 5, n = 3149), αSyt1-CypHer (**D**) (N = 5, n = 5698) and synaptopHluorin (**F**) (N = 5, n = 5835) labelling experiments. (Scale bars 8 µm; two-sample t-test: *** p<0.001). Error bars indicate standard error of the mean.

We found that synaptic terminals marked by αSyt1-CypHer were heterogeneously distributed in hippocampal neuronal cultures (Figure 3C). Here the number of neighbours of individual terminals was also highly correlated with their amplitudes of stimulation dependent changes in αSyt1-CypHer fluorescence (Figure 3D; table 1; two-sample t-test: p<0.001). This confirms that synapses with large recycling pool sizes have a large number of neighbours.

**Table 1:** Correlation coefficient and effect sizes of individual experiments of the three different functional labelling approaches.

		FM 1–43	αSyt1-CypHer	spH
**Number of neighbours - pool size**	**Spearman's ** ***ρ***	0.97±0.03,	0.88±0.08,	0.74±0.08,
		p<0.01	p<0.01	p<0.01
	**Cohens ** ***d***	1.05±0.11	1.23±0.30	0.79±0.15
**Number of neighbours - mean pool size of neighbours**	**Spearman's ** ***ρ***	0.95±0.02,	0.92±0.07,	0.77±0.06,
		p<0.01	p<0.01	p<0.01
	**Cohens ** ***d***	1.80±0.16	1.59±0.45	0.81±0.23
	**Number of experiments**	30	5	5

Effect size classes (Cohen 1988): *d* = 0.2 small effect, *d* = 0.5 medium effect, *d* = 0.8 strong effect. The number of neighbours has a strong effect (Cohens *d*> = 0.8 in all approaches) on the size of the recycling pool. Errors indicate standard error of the mean.

In an overexpression approach we chose to transfect hippocampal neurons with synaptopHluorin (spH) which also can be used to define functional synaptic boutons [31] and marks the amount of exocytosed vesicles [25]. While here the analysis was restricted to presynapses of transfected neurons, the main findings were preserved. Neighbour numbers of synaptopHluorin positive boutons were heterogeneously distributed (Figure 3E). Synapses with large amount of vesicles released upon stimulation and thus large recycling pools had significantly more neighbours as synapses with small stimulation dependent fluorescence increases (Figure 3F; table 1; two-sample t-test: p<0.001).

## Discussion

The release characteristics of individual synapses provide the basis for network phenomena such as long-term memory formation and learning. The regulation of the individual synapses parameters has therefore been investigated intensively. For example, synaptic release probability and synapse size increased after complete block of fast voltage-gated sodium channels with tetrodotoxin [6]. Accordingly, a chronic blockade of cortical culture activity increased the amplitude of miniature excitatory postsynaptic currents (mEPSCs) [44] without changing their kinetics [45]. Block of activity had no influence on the frequency of miniature postsynaptic currents [44] that however are correlated to the size of an individual synapses recycling pool [7]. Reactivation of a neural circuit by releasing it from tetrodotoxin blockade resulted in an increase of the frequency of AMPAR-mediated mEPSCs [4]. Accordingly, increased synaptic activity decreased the synaptic release probability and size of the postsynaptic response to a single quantum of neurotransmitter [45], [46].

Besides these functional parameters, activity-dependent changes of the spatial distribution of synapses within networks were also analysed. The number of functional excitatory synapses in the CA3 area of the developing hippocampus was found to be increased after blockade of spontaneous network activity [3]. Also, it was shown that reduction of network activity in mature neural circuit promotes reorganization of these circuits via NR2B subunit-containing NMDA-type glutamate receptors, which mediate silent synapse formation [4]. On the single synapse level, it has been shown recently that global blockage of activity also leads to a decreased ability of synapses to maintain their size [47]. On the other hand, under intense stimulation the overall number of synapses decreases while in some areas synapses are preserved [2], whereby synapse cluster formation is critically affected by neuronal activity [5].

These results show the diverse reactions of neural networks following stimulation and non-stimulation. In either case, the integrated individual synaptic changes in groups of functionally related neurons result in network plasticity. This network plasticity implies synaptic rewiring and synaptic weight changes [1], which in turn might regulate not only the heterogeneity in size but also the heterogeneity in spatial distribution of synapses.

Indeed, a heterogeneous spatial distribution of synapses can be observed in acute slices and *in vivo* recordings. Even though these studies did not specifically focus on synapse distribution, beautiful examples can be found in their figures: the mere visual impression is sufficient to spot a heterogeneity in acute hippocampal slice preparations (see Figure 3 in [48] or Figure 1 in [49]), and in an *in vivo* example (see Figure 1 in [50]).

To actually analyze the spatial distribution and synaptic release parameters, we used an established rat hippocampal tissue culture system. In this system, neurons create a network similar to an *in vivo* network [2], [51], [52]. Importantly, fundamental neuronal functions such as LTD and LTP are shown to be preserved [53], [54], [55]. By staining the recycling vesicle pool by performing FM styryl dye turnover experiments, we found a heterogeneous distribution of the number of neighbours for each bouton (see Figure 1C), resulting in areas of varying synapse densities. We next addressed the determinants of vesicle release of the individual synapses to find a connection between the number of adjacent synapses and individual synaptic release characteristics. Here, a strong correlation between the number of adjacent synapses and the recycling pool size was found (see Figure 2C). To validate these findings, we used two independent functional labelling assays and obtained similar results. Other synaptic parameters reported in previous studies [6], [9], [10], [18], [19], [41], [42] were also analyzed and found to agree with our results. Synapses with many neighbours and therefore large recycling pools exhibited a high release probability and synapse diameters (Figure S1). This is likely due to their larger active zones and readily releasable pool [26].

Considering that release probability and the formation of synapse clusters is inversely regulated by synaptic activity [5], [6], one would expect areas of high synaptic activity such as synapse clusters would be composed of synapses with small recycling pool sizes. In contrast, our results show that a common feature of synapses with large recycling pools was their location in close proximity to other synapses with large vesicle pools (Figure 2C).

We thus report a high release probability in synapse clusters which previously has remained elusive. We showed that this is not only due to the mere spatial accumulation of synapses [5], but that neurotransmitter release in synapse clusters is also enhanced by the large recycling pool size of synapses. As evident from the heterogeneous distribution of release probabilities in our model system, the 10% of synapses with largest recycling pools account for 25% of the evoked vesicular release (Compare distribution in Figure S1B). Consequently, the accumulation of synapses with large recycling pools in clusters results in the formation of “hot spots of synaptic activity” (Figure 2).

Our results might have functional implications within neural networks. As we found clusters of synapses with large recycling pools, these might represent strengthened connections due to Hebbian learning. In this model a successful synapse is rendered strong and all other synapses relatively weak [56], [57]. The positive reinforcement leads to a synaptic landscape where a few strong synapses reside over a background of weak synapses [57]. On the other hand, negative feedback in terms of depressing inadequate synapses together with the assumption that neuronal activity propagates only through the network's strongest synaptic connections is sufficient for adaptive learning [57], [58]. Other than Hebbian learning, this model requires synaptic connections transmitting information from one to the next neuron very effectively [57]. Both views fit well to the previously described heterogeneity in recycling pool sizes of synapses. To this view, our findings add the heterogeneous spatial distribution of synapses as an important parameter.

Given our results, synapse clusters can be considered as nodes of high transmission probability. This has implications on a more macroscopic view of neural network analysis. Recent studies found neuronal avalanches as a mode of activity that satisfies the competing demands of network stability and transmission efficiency [59]. This transmission was found to be associated with maximal information transfer and thus a high efficacy of neuronal information processing [59], [60]. Together with the fact that neural network activity scales with synapse density [61] and as synapses are the early input side of information processing, synapse clusters may be nodes of high transmission likelihood in neuronal avalanches and will shape at least some cortical activation patterns. Additionally, failure in synaptic transmission might be compensated more easily in synapse clusters, as nearby synapses are likely to be connected to the same dendrite [5].

Furthermore, if clusters are treated as nodes within networks, they might support synchronous processing [62], [63] or efficient information exchange [64]. In neural networks, action potentials propagate in synchronized patterns through several synaptic stages without much attenuation [59], [65], [66], [67]. “Hot spots of activity” like synapse clusters could be able to contribute to this observed low attenuation.

Finally, considering that adjacent synapses share vesicles [21] and that vesicle sharing can lead to the formation of vesicular superpools spanning multiple terminals [22], it is tempting to speculate that this form of intracellular and also extracellular [68] inter-synaptic communication is enhanced especially in synapse clusters or contributes to their formation. As we have found the number of synaptic neighbours to be continuously distributed in our model system, superpooling of synapses could be regarded as the highest level of a common organizational principle: an increasing recycling pool size of individual synapses is accompanied by their accumulation to synapse clusters.

Origin and regulation of synapse clusters could define their role in development and maintenance of functional networks. It thus will be interesting to elucidate specific aspects, including the contribution of excitatory and inhibitory terminals or the influence of neurotrophic factors on recycling pool size and synapse clustering.

## Supporting Information

Figure S1 Determination of single synaptic vesicle fluorescence, correlation between recycling pool size and quanta released by 600 APs, release probability following stimulation of 20 AP and correlation between synapse diameter and recycling pool size. A, Histogram of ΔFtot values of 181 boutons loaded with 20 APs. Solid black line is a multiple gaussian fit with peaks at almost equal 20 a.u. intervals. Single gaussians with equal widths are represented by red dotted lines. To determine the number loaded quanta the mean interval between the centers of the gaussian peaks were used. Using this approach we determined the mean fluorescence of a single vesicle to 20.02±1.91 a.u. (n = 613; N = 3; Coefficient of determination R2 = 0.994±0.01). B, Correlation of exocytosed quanta with recycling pool size during stimulation with 600 APs at 30 Hz and distribution of recycling pool size for this measurement series (Cohens d = 9.84, Spearman's ρ = 0.96, p<0.001). The mean value of the recycling pool size (137 vesicles) is indicated by the red dotted line. C, Distribution of fused vesicles and release probabilities following stimulation of 20 APs. The histogram is well fitted by Γ (2, λ) with λ = 8.32 (dotted line). D, Correlation between synapse diameter and recycling pool size (n = 268, N = 5). The mean and standard deviation of the synapse diameter was 882.09 nm±249.92 nm. Data was grouped in bins and averaged. With increasing synapse diameter the recycling pool size increases (Wilcoxon rank sum test: ** p<0.01, *** p<0.001; Spearman's ρ = 0.51, p<0.001; Cohens d = 2.49). Error bars indicate standard deviations for synapse diameter and recycling pool size, respectively.
(1.25 MB DOC)

Figure S2 Fluorescence difference image of images before and after complete destain (twofold stimulation with 900 APs at 30 Hz). Synaptic vesicles were loaded with the styryl dye FM 1–43 (1200 AP, 40Hz). This difference-image was used to generate image in Fig. 2A. (Scale bar 8 µm).
(0.97 MB DOC)
