# Supplementary figures and images for: Synapse Clusters Are Preferentially Formed by Synapses with Large Recycling Pool Sizes
Source: PLoS One. 2010 Oct 20;5(10):e13514. doi: 10.1371/journal.pone.0013514 (PMC2958124; doi:10.1371/journal.pone.0013514)

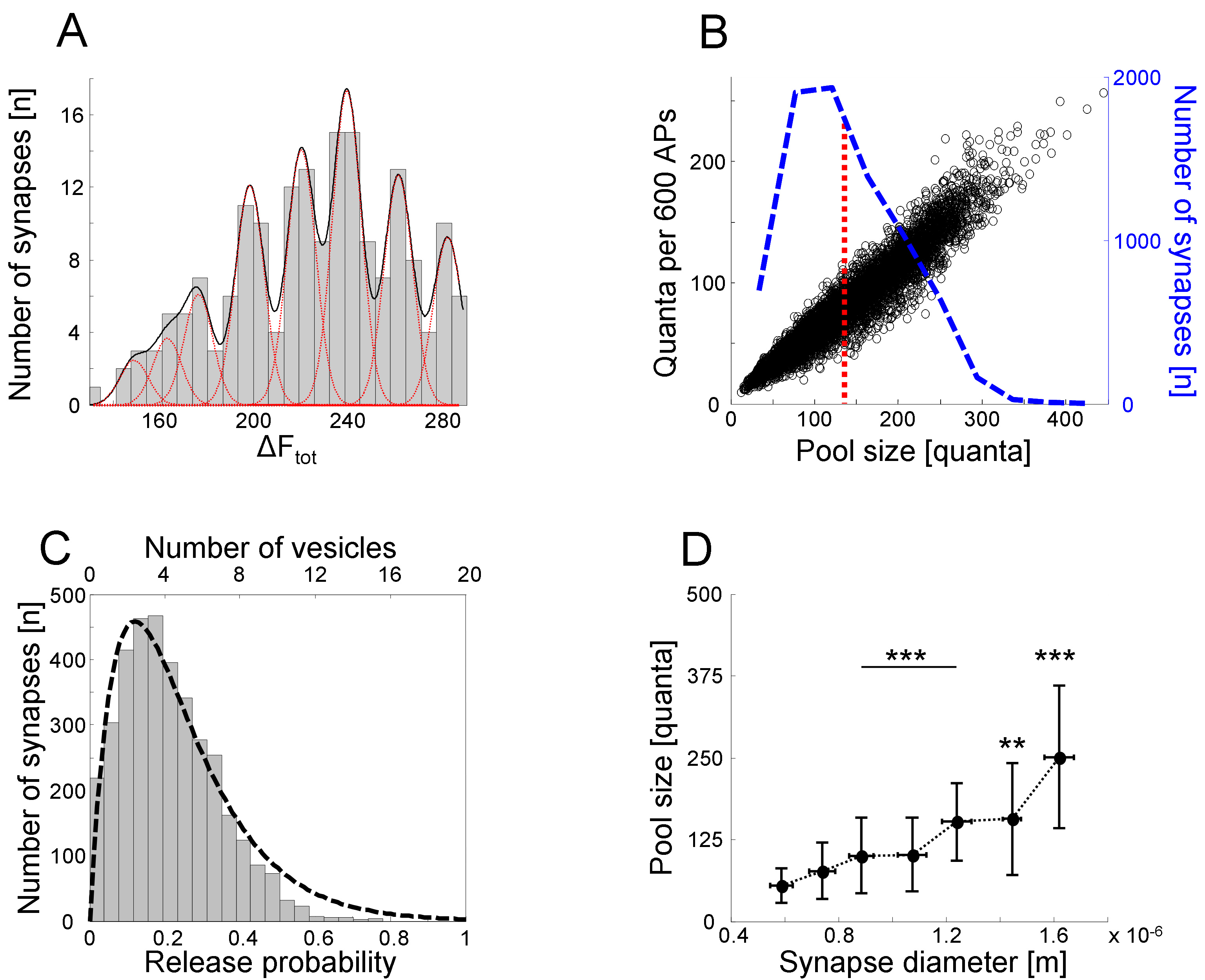

Supplement: Figure S1 — Determination of single synaptic vesicle fluorescence, correlation between recycling pool size and quanta released by 600 APs, release probability following stimulation of 20 AP and correlation between synapse diameter and recycling pool size. A, Histogram of ΔFtot values of 181 boutons loaded with 20 APs. Solid black line is a multiple gaussian fit with peaks at almost equal 20 a.u. intervals. Single gaussians with equal widths are represented by red dotted lines. To determine the number loaded quanta the mean interval between the centers of the gaussian peaks were used. Using this approach we determined the mean fluorescence of a single vesicle to 20.02±1.91 a.u. (n = 613; N = 3; Coefficient of determination R2 = 0.994±0.01). B, Correlation of exocytosed quanta with recycling pool size during stimulation with 600 APs at 30 Hz and distribution of recycling pool size for this measurement series (Cohens d = 9.84, Spearman's ρ = 0.96, p<0.001). The mean value of the recycling pool size (137 vesicles) is indicated by the red dotted line. C, Distribution of fused vesicles and release probabilities following stimulation of 20 APs. The histogram is well fitted by Γ (2, λ) with λ = 8.32 (dotted line). D, Correlation between synapse diameter and recycling pool size (n = 268, N = 5). The mean and standard deviation of the synapse diameter was 882.09 nm±249.92 nm. Data was grouped in bins and averaged. With increasing synapse diameter the recycling pool size increases (Wilcoxon rank sum test: ** p<0.01, *** p<0.001; Spearman's ρ = 0.51, p<0.001; Cohens d = 2.49). Error bars indicate standard deviations for synapse diameter and recycling pool size, respectively. (1.25 MB DOC) [file pone.0013514.s001.doc]

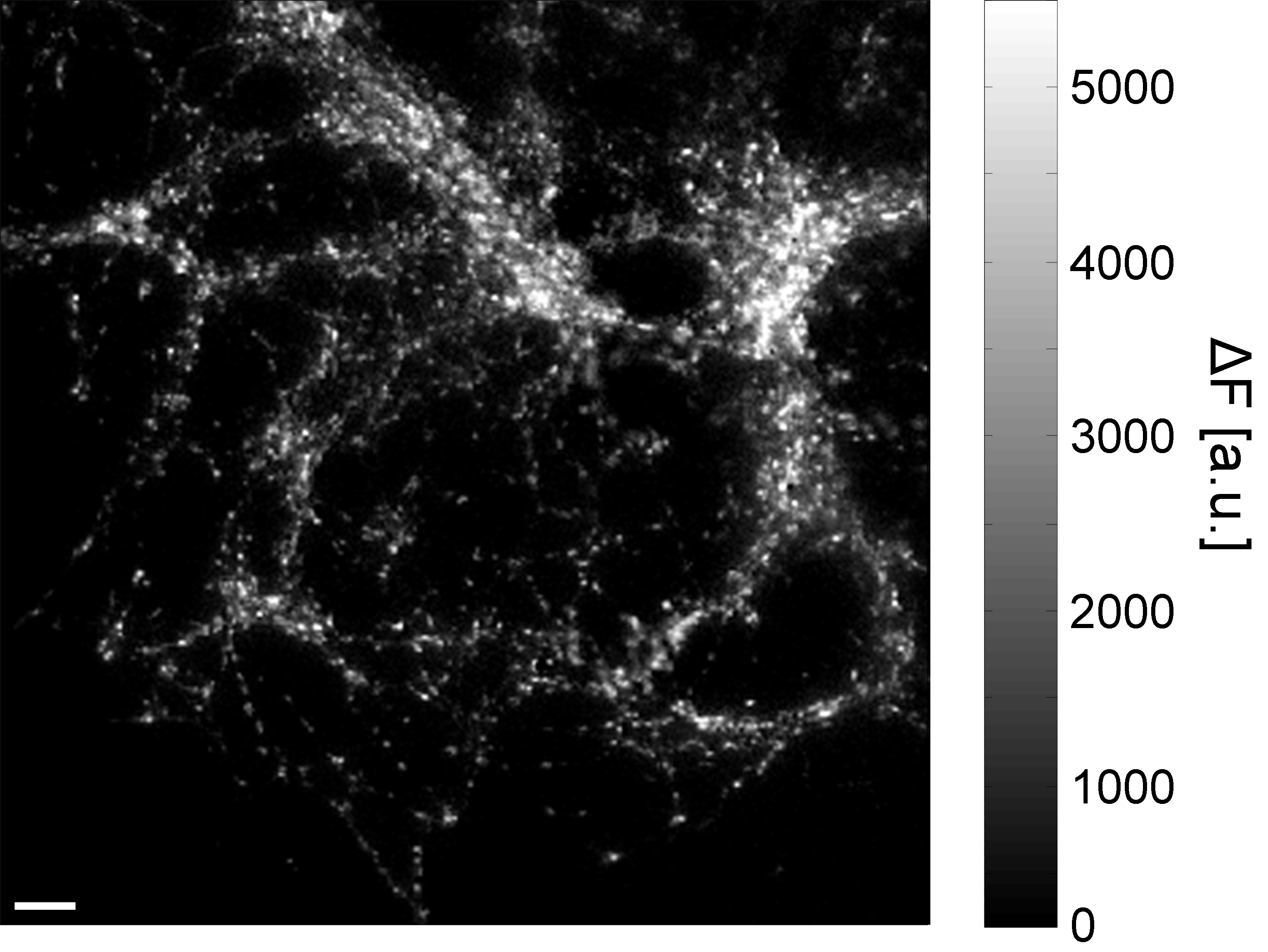

Supplement: Figure S2 — Fluorescence difference image of images before and after complete destain (twofold stimulation with 900 APs at 30 Hz). Synaptic vesicles were loaded with the styryl dye FM 1–43 (1200 AP, 40Hz). This difference-image was used to generate image in Fig. 2A. (Scale bar 8 µm). (0.97 MB DOC) [file pone.0013514.s002.doc]
